# Editorial

**DOI:** 10.1049/enb2.12006

**Published:** 2021-03-25

**Authors:** Richard I. Kitney, Chueh Loo Poh

**Affiliations:** ^1^ Imperial College London London UK; ^2^ National University of Singapore Singapore

1

We start the fifth volume of *Engineering Biology* at a time that it is becoming increasingly clear that the engineering biology field is likely to have a very significant impact on the growth of the international bioeconomy and the drive towards low carbon. In addition, the field is predicted to have significant impact in biomedicine—for example, in vaccine development and the way in which vaccines are manufactured, with the contribution of Ginkgo Bioworks to the development of the Moderna vaccine for COVID‐19 a timely demonstration of this. There are also many other areas where engineering biology is being applied to replace existing industrial processes and products and we are delighted to have published some of this work in the journal over the last year including the development of synthetic spider silk at The University of Manchester, which is already having applications in a number of areas, and the application of machine learning‐driven protein engineering by LabGenius for computational drug discovery. At the end of the year, we also published the popular opinion piece ‘Synthetic biology, engineering biology, market expectation’ by Lionel Clarke, Co‐Chair of the UK Engineering Biology Leadership Council and Director of BionerG, in which he discusses the differences between the terms ‘engineering biology’ and ‘synthetic biology’ and their role in the market ‘ecosystem’. In this first issue of our fifth volume, we are pleased to present a review of the optimisation of protein synthesis in cell‐free systems—an area that has a number of applications; and the case study ‘Responsible Innovation: its role in an era of technological and regulatory transformation’.

We would like to take this opportunity to thank all of the contributors to the journal: the authors from academia and industry for sharing their interesting developments and insights; the reviewers for the time they have taken to assess and provide valuable feedback on the submissions, and the Associate Editors for their work to uphold the high standards of the journal. We are very grateful for the efforts of all involved and we are continuing our work with our international editorial board to build the journal into a key source of information for all aspects of engineering biology.

*Engineering Biology* has a deliberately wide scope so that it can capture not only original research, but also industrial developments (through contributions by industrial companies); opinion pieces on the grand challenges, policies, new projects and developments in the field; and invited mini reviews of progress in important areas. This year the publisher of *Engineering Biology*, The Institution of Engineering and Technology (IET), has partnered with Wiley to publish its journals, and we believe that this will give *Engineering Biology* an even greater outreach and range of readership. Please do get in touch with us at theiet_enb.office@wiley.com if you are interested in submitting an article or have an idea for a special issue. You can also find more information about the journal and how to submit at http://wileyonlinelibrary.com/iet‐enb. We are looking forward to working with the engineering biology community over the coming year to publish more of the exciting developments in this area.

